# Contribution of CXCL12 secretion to invasion of breast cancer cells

**DOI:** 10.1186/bcr3108

**Published:** 2012-02-07

**Authors:** Pamela J Boimel, Tatiana Smirnova, Zhen Ni Zhou, Jeffrey Wyckoff, Haein Park, Salvatore J Coniglio, Bin-Zhi Qian, E Richard Stanley, Dianne Cox, Jeffrey W Pollard, William J Muller, John Condeelis, Jeffrey E Segall

**Affiliations:** 1 Department of Anatomy and Structural Biology, Albert Einstein College of Medicine, 1301 Morris Park Ave., Bronx, NY, 10461, USA; 2 Gruss Lipper Center for Biophotonics, Albert Einstein College of Medicine, 1301 Morris Park Ave., Bronx, NY, 10461, USA; 3 Department of Developmental and Molecular Biology, Albert Einstein College of Medicine, 1300 Morris Park Ave., Bronx, NY, 10461, USA; 4 Department of Biochemistry, McGill University, McIntyre Medical Building, 3655 Promenade Sir William Osler, Room 802, Montreal, QC, H3G 1Y6, Canada; 5 Center for the Study of Reproductive Biology and Women's Health, Albert Einstein College of Medicine, 1300 Morris Park Ave., Bronx, NY 10461, USA

## Abstract

**Introduction:**

Neu (HER2/ErbB2) is overexpressed in 25% to 30% of human breast cancer, correlating with a poor prognosis. Researchers in previous studies who used the mouse mammary tumor virus Neu-transgenic mouse model (MMTV-Neu) demonstrated that the Neu-YB line had increased production of CXCL12 and increased metastasis, whereas the Neu-YD line had decreased metastasis. In this study, we examined the role of increased production of CXCL12 in tumor cell invasion and malignancy.

**Methods:**

We studied invasion in the tumor microenvironment using multiphoton intravital imaging, *in vivo *invasion and intravasation assays. CXCL12 signaling was altered by using the CXCR4 inhibitor AMD3100 or by increasing CXCL12 expression. The role of macrophage signaling *in vivo *was determined using a colony-stimulating factor 1 receptor (CSF-1R) blocking antibody.

**Results:**

The Neu-YD strain was reduced in invasion, intravasation and metastasis compared to the Neu-YB and Neu deletion mutant (activated receptor) strains. Remarkably, in the Neu-YB strain, *in vivo *invasion to epidermal growth factor was dependent on both CXCL12-CXCR4 and CSF1-CSF-1R signaling. Neu-YB tumors had increased macrophage and microvessel density. Overexpression of CXCL12 in rat mammary adenocarcinoma cells increased *in vivo *invasion as well as microvessel and macrophage density.

**Conclusions:**

Expression of CXCL12 by tumor cells results in increased macrophage and microvessel density and *in vivo *invasiveness.

## Introduction

Neu (HER2/ErbB2) is overexpressed in 25% to 30% of human breast cancer, correlating with a poor prognosis [1]. Neu is a member of the ErbB family of receptor tyrosine kinases, which are important mediators of signal transduction for proliferation, survival, apoptosis, motility and invasion of cells. The ErbB receptors, consisting of ErbB1 (epidermal growth factor receptor (EGFR)), Her2/Neu (ErbB2), ErbB3 and ErbB4, can homodimerize and heterodimerize, mediating ligand specificity and various signal transduction pathways [2]. At low expression levels, Neu is unlikely to homodimerize [3]; however, it is the preferred binding partner for the other ErbB receptor tyrosine kinases and mediates the activation of potent signal transduction pathways [4,5].

At high expression levels, Neu can homodimerize [6,7], and the correlation of high levels of expression with poor prognosis and clinical significance as a pharmacological target has made the Neu receptor and its contributions to metastasis and tumorigenesis important areas of study. Because of its clinical significance, the Her2/Neu receptor has been the focus of studies aimed at pharmacologically inhibiting its function. Trastuzumab (Herceptin; Genentech, South San Francisco, CA, USA), a human mAb, has been used to treat patients with Her2-positive breast cancer [8]. However, the development of drug resistance to trastuzumab treatment [9] underscores the necessity to continue to investigate new ways to inhibit the receptor pharmacologically. To study the Neu receptor *in vivo*, a small deletion mimicking that found in patient tumors [10], was made in the extracellular domain, and this construct (termed "Neu deletion mutant (activated receptor)," or Neu-NDL) was expressed by the mouse mammary tumor virus (MMTV) promoter in transgenic mice [10,11]. A series of mutations of Neu-NDL were made in which the major C-terminal phosphorylated tyrosine residues were mutated to phenylalanine, after which individual tyrosines were added back and referred to as YA (1,028), YB (1,144), YC (1,201), YD (1,227) and YE (1,253) [12].

Using these add-back mutants, we studied the contributions of the tyrosine sites to tumorigenesis and lung metastasis in transgenic mice. We found that the YA site impaired transformation and/or tumorigenesis, the YB site increased and the YD site decreased metastasis, whereas the other add-back mutants exhibited metastasis rates similar to that of Neu-NDL [12-14]. Metastasis is a series of steps involving tumor growth, angiogenesis, motility in the tumor microenvironment, invasion, intravasation, extravasation and growth of metastases at a distant site such as the lungs [15]. We chose to study how the YB and YD sites diverge in their contributions to early stages of metastasis by using the Neu-transgenic mouse model and *in vivo *assays for tumor cell motility, invasion and intravasation.

It has previously been shown on the basis of microarray and ELISA that the YB line tumors express more CXCL12 (stromal cell-derived factor 1) than the other lines [14]. CXCL12 binds to the G protein-coupled receptor CXCR4, which is often overexpressed in breast cancer and has been correlated with poor clinical outcome [16,17]. CXCL12-CXCR4 signaling has been shown to play a role in tumor growth, invasion, angiogenesis and bone marrow cell recruitment [18-23]. Recent studies of autocrine CXCL12 signaling have indicated that it can induce the differentiation of monocytes into a distinct population of proangiogenic, immunosuppressive macrophages in the tumor microenvironment [24]. The results of these studies indicate that overexpression of CXCL12 in the tumor microenvironment may alter invasive capacity, as well as the tumor-associated immune cells that are recruited to tumors. CXCL12 overexpression has been linked to increased metastasis and poor prognosis [25]. Targeting the CXCL12-CXCR4 signaling pathway has also been studied in breast cancer treatment [26,27]. In this article, we report that increased expression of CXCL12 by breast cancer cells can lead to enhanced *in vivo *invasion and increased macrophage and microvessel density.

## Materials and methods

### Animal models and cell lines

The experimental procedures involving all mice were conducted in accordance with National Institutes of Health regulations on the care and use of experimental animals. This mouse study was approved by the Albert Einstein College of Medicine animal use committee. FVB mice transgenic for Neu and containing a small activation deletion (Neu-NDL), as well as the add-back mutants Neu-YB and Neu-YD [10,11], driven by the MMTV long terminal repeat were studied. Mice were followed for tumor growth and were used for experiments when the largest tumor reached 2 cm in diameter. Tumor volume was calculated as length × (width^2 ^÷ 2). For intravital imaging, the strains were crossed with a strain expressing cyan fluorescent protein (CFP) using the mammary gland-specific MMTV promoter (MMTV-iCre-CAG-CAC-ECFP) as previously described [28,29]. Xenograft tumors were made by detaching rat mammary adenocarcinoma (MTLn3) tumor cells with a solution containing PBS and 2 mM ethylenediaminetetraacetic acid (EDTA) and then injecting 1 × 10^6 ^cells, which were resuspended in PBS with 0.35% BSA (wt/vol), down from the heads to the right fourth mammary fat pads of 5- to 7-week-old female severe combined immunodeficient mice (National Cancer Institute, Frederick, MD, USA). For the *in vivo *invasion assay, xenograft tumors were grown until they reached 1.5 cm in diameter (about 4 weeks).

For culturing, primary tumors were chopped into small pieces, washed in PBS and dissociated in PBS with collagenase IV (final concentration of 6 mg/ml, C5138; Sigma-Aldrich, St Louis, MO, USA), hyaluronidase (final concentration of 1 mg/ml, H3506; Sigma-Aldrich) and DNase I (final concentration of 0.25 mg/ml, D5025-15KU; Sigma-Aldrich) for 30 minutes with continuous agitation at 37°C. Following digestion, samples were washed twice in sterile PBS and plated in DMEM/Ham's F-12 nutrient mixture (MT10092CV; Fisher Scientific, Pittsburgh, PA, USA) supplemented with 10% fetal bovine serum (FBS) (100-106; Gemini Bio-Products, West Sacramento, CA, USA) and 0.5% penicillin-streptomycin 100× solution (15140-122; Gibco/Life Technologies, Grand Island, NY, USA), 1 μg/ml hydrocortisone (h4001; Sigma-Aldrich), 10 μg/ml insulin (I5523; Sigma-Aldrich), 1 nM cholera toxin (C8052; Sigma-Aldrich), nonessential amino acids and 10 nM epidermal growth factor (EGF) (Life Technologies, Carlsbad, CA, USA).

To overexpress mouse CXCL12, a pCMVSport6-CXCL12 plasmid (accession numbers BC006640 and EMM1002-4021797; Open Biosystems/Thermo Scientific, Lafayette, CO, USA) was blunt-subcloned into the NotI restriction enzyme site of the pQCXIP vector and confirmed by plasmid sequencing. Retrovirus was generated by transfection of 293GP cells using Lipofectamine 2000 transfection reagent (11668-019; Invitrogen) with 12 μg of plasmid DNA and 4 μg of VSV-G plasmid (vesicular stomatitus virus G protein). After 48 hours, supernatants were collected and used to transduce MTLn3-GFP mammary adenocarcinoma cells [30] in the presence of 4 μg/ml polybrene. All MTLn3 cell lines were grown in α-minimum essential medium (α-MEM) supplemented with 5% FBS and 0.5% penicillin-streptomycin. Transduced cells were selected with 1 μg/ml puromycin. To confirm expression of CXCL12, MTLn3 cells were seeded into six-well dishes in triplicate and supernatants were collected 16 hours after the cultures were confluent. Cells were counted at the time of collection to quantify the concentration in picograms per milliliter for 1 × 10^6 ^cells. To evaluate CXCL12 secreted *in vitro *by the Neu primary tumor cell lines, dissociated tumor cells were seeded at equal density into six-well dishes and supernatants were collected 16 hours after the cells were confluent. Samples were spun down to remove debris, frozen on dry ice and stored at -80°C. CXCL12 was measured in the supernatant by ELISA (DY460; R&D Systems, Minneapolis, MN, USA), with triplicate measurements done for each sample. The concentration of CXCL12 was calculated in picograms per milliliter using a standard curve from serial diluted CXCL12 provided in the ELISA kit. MTLn3-GFP cells transduced with JP1520 vector expressing CXCR4 were kindly provided by Dr Lorena Hernandez [31].

### Immunohistochemistry

Tumors were fixed in 10% buffered formalin for a minimum of 24 hours, paraffin-embedded and sectioned for H & E staining. Tumors were also fixed in periodate-lysine-2% paraformaldehyde-0.05% glutaraldehyde (PLPG) [32] at 4°C overnight, then stored in 70% ethanol at 4°C until embedded in paraffin. PLPG fixed tumors were stained with F4/80 antibody at 1:50 dilution to identify macrophages as previously described [33]. To quantify F4/80 staining, we chose ten random fields of healthy tumor parenchyma and counted the number of F4/80-stained macrophages using a 40× lens objective for three tumors per strain. We found that formalin-fixed tumor vessels were poorly stained by anti-CD34 antibodies; therefore, to stain tumor vasculature, formalin-fixed, paraffin-embedded tumor sections were stained with a rat anti-mouse endomucin (V.7C7) mAb (sc-65495; Santa Cruz Biotechnology, Santa Cruz, CA, USA) at 1:50 dilution. To quantify endomucin staining, ten random fields of healthy tumor parenchyma were counted for endomucin-stained vessels using a 20× lens objective for three tumors per strain. To detect tumor lymphatic vessels, formalin-fixed, paraffin-embedded tumor sections were stained with a rabbit anti-mouse lymphatic vessel endothelial hyaluronan receptor pAb (ab14917; Abcam, Cambridge, MA, USA) at 1:200 dilution. Anti-CXCR4 antibody was used at 1:300 dilution (ab7199; Abcam).

To quantify lung metastasis in the Neu-transgenic tumors, lung samples were fixed in 10% neutral formalin buffer, embedded in paraffin, sectioned at 5 μm and stained with H & E. H & E-stained sections spaced 250 μm apart were taken from the whole lung sample, and all micrometastases were counted in every section using a light microscope with a 10× lens objective. The efficiency of lung metastasis was expressed in total number of metastases in all lung sections for each animal with metastases. To quantify lung metastases in the MTLn3 pQCXIP and pQCXIP-CXCL12 tumors, lungs were removed after 4 weeks when the tumors had reached 1.5 cm in diameter and fixed in 10% neutral formalin buffer, embedded in paraffin, sectioned and stained with H & E to quantify the total number of lung metastases in all lobes per section on a light microscope with a 10× lens objective.

### *In vivo *invasion assay

Tumors were allowed to reach 2-cm diameter (Neu-transgenic strains) or 1.5-cm diameter (MTLn3 xenografts) for use in the *in vivo *invasion assay. The collection of the invasive cell population from the primary tumors into microneedles containing matrix and chemoattractant using an *in vivo *invasion assay has been described previously [34]. The mice were anesthetized with isoflurane (Aerrane; Baxter Healthcare Corp, Deerfield, IL, USA) during the course of the assay. Briefly, tumors were first penetrated with 33-gauge Hamilton needles (14-815-423; Fisher Scientific), then these needles were replaced with ones filled with L15 medium and 0.35% BSA containing 10% BD Matrigel™ Basement Membrane Matrix (356234; BD, Franklin Lakes, NJ, USA) with or without chemoattractant. Invasive cells were collected for 4 hours, then the contents of the needles were extruded onto a coverslip by using 0.5 μg/ml 4',6-diamidino-2-phenylindole in PBS to quantify the number of invasive cells with an Olympus IX70 inverted microscope with a 10× lens objective and a 0.30 numerical aperture (NA) (Olympus America, Melville, NY, USA). The chemoattractant EGF (53003018; Gibco/Life Technologies) was used at a concentration of 25 nM. To inhibit the CXCR4 receptor, the inhibitor AMD3100 (A5602; Sigma-Aldrich) was used at a concentration of 0.5 μM. To block and/or neutralize the colony-stimulating factor 1 receptor (CSF-1R), a purified anti-mouse CSF-1R mAb, AFS98 [35], kindly provided by Dr Richard Stanley was used at a concentration of 15 μg/ml. As a control antibody, a mouse immunoglobulin G (IgG) antibody was used at the same concentration used for the experimental CSF-1R antibody (012-000-007; Jackson ImmunoResearch, West Grove, PA, USA).

### Wound healing assay

Primary tumor cells were cultured for 1 week, passaged into a new 10-cm culture dish and allowed to grow to confluence. We removed EGF from the media 24 hours prior to performing wound healing assays. Cultures for each of the strains were starved in DMEM/Ham's F-12 nutrient mixture and 0.7% BSA (wt/vol) for 4 hours, then a grid was drawn on the plate for reference and four scratches were introduced using a pipette tip. Images were taken at time 0, and cultures were treated with buffer and 5 nM EGF. The Neu-YB strain was also treated with 5 nM EGF + 100 nM AMD3100, 5 nM EGF + 1 nM CXCL12 and 1 nM CXCL12. The wound was allowed to heal for 12 hours, and images of the same field were taken. Analysis was done using TScratch software as previously described [36], and the percentage of the wound area closed was calculated.

### Chemotaxis assays

Murine bone marrow-derived macrophages (BMMs) were isolated as described previously [37] and were grown in α-MEM containing 15% FBS, 360 ng/ml recombinant human CSF-1 (Chiron Corp, Emeryville, CA, USA) and antibiotics. Cell migration was measured using a transmigration chamber assay with 8-μm pore size inserts (BD Falcon, Franklin Lakes, NJ, USA) according to the manufacturer's instructions. Briefly, the inserts were placed into 24-well plates containing α-MEM in the presence or absence of 50 ng/ml CXCL12. Cells (*n *= 1 × 10^6^) were then loaded onto the inserts and incubated at 37°C for 4 hours. Cells that had migrated through the inserts were counted using phase microscopy, and the average number of cells in five different fields was calculated. For MTLn3-GFP-CXCR4 cells, a 48-well microchemotaxis chamber (Neuro Probe, Inc, Cabin John, MD, USA) was used as described previously [38]. Briefly, cells were starved for 3 hours in L15 medium supplemented with 0.35% BSA (L15B) at 37°C. Two hours before microchemotaxis chamber assembly, an 8-μm pore filter (Neuro Probe, Inc) was coated with 3.53 mg/ml rat tail collagen type I (354249; BD) in Ca^2+^- and Mg^2+^-free Dulbecco's PBS. After starvation, cells were detached using PBS-EDTA and 30,000 cells per well were loaded into the top wells of the chamber in L15B medium. Cells were allowed to migrate for 4 hours at 37°C, with the bottom wells filled with CXCL12 diluted in L15B or with L15B alone. The filter was then taken out and fixed in 10% paraformaldehyde for 1 hour, nonmigrating cells were removed from the top surface of the filter and the remaining cells on the lower surface were stained overnight with hematoxylin (CS402-1D; Fisher Scientific). The membrane was washed in deionized water, and migrated cells were counted using a light microscope.

### Immunofluorescence microscopy

Live, nonpermeabilized murine BMMs were stained with 1:250 anti-CXCR4 antibody (ab7199; Abcam) and Alexa Fluor 568 dye donkey anti-rabbit IgG. Images were taken using the Plan Apochromat (Plan Apo) 60× oil immersion lens/1.40-NA phase 3 objective of an Olympus IX71 microscope equipped with a charge-coupled device camera (Olympus America).

### Quantitative RT-PCR

mRNA was isolated from cells in culture, and 100 ng of cDNA were used for quantitative RT-PCR. Specific primers for mouse CXCR4 (forward: 5'-TGGTGTTTCAATTCCAGCAT-3' and reverse 5'-CGATGCTCTCGAAGTCACAT-3') were obtained from RealTimePrimers.com (mouse chemokine (C-X-C motif) receptor 4, VMPS-1496; Real Time Primers LLC, Elkins Park, PA, USA) and used with SYBR Green PCR Master Mix (SuperArray PA-012; SABiosciences, Frederick, MD, USA).

### Blood burden assay

Mice were anesthetized with isoflurane, and blood was taken from the right atrium by cardiac puncture using a 25-gauge needle and a 1-ml syringe coated with and containing 0.1 ml of heparin. Blood (0.5 to 1 ml) was drawn and plated into 10-cm-diameter dishes filled with DMEM/Ham's F-12 nutrient mixture and 20% FBS. The next day the plates were replaced with fresh medium. After 7 days, the number of individual cells in the dish were counted. Tumor blood burden (tumor cells per milliliter of blood) was calculated as the total number of tumor cells in the dish divided by the volume of blood taken.

### Intravital imaging

Animals with an average tumor diameter of 2 cm were placed under isoflurane anesthesia, and skin flap surgery was performed to expose the tumor. These animals were placed on an inverted microscope while under continuous anesthesia, and tumor cells were imaged using a Bio-Rad Radiance 2000 MP multiphoton microscope (Bio-Rad Laboratories, Hercules, CA, USA) or on an Olympus FluoView FV1000-MPE multiphoton laser scanning microscope (Olympus America). The CFP fluorescence was measured at a wavelength of 880 nm using a Plan Apo 20× water immersion lens/0.75-NA objective with a 3× zoom or a Plan Apo 25× water immersion lens/1.05-NA objective with a 2.3× zoom. Time-lapse imaging was done over 30 minutes with 2-minute intervals to collect a 100-μm stack. Motility was assessed using ImageJ software (National Institutes of Health, Bethesda, MD, USA), and the average total cell motility was quantified per 50-μm Z-stack (five sections imaged at 10-μm intervals). A motile cell was defined as any protrusion at least one-half cell diameter in length.

## Results

### Neu-YD strain exhibited decreased spontaneous invasion, intravasation and metastasis compared to Neu-NDL and Neu-YB strains

**Figure 1:**
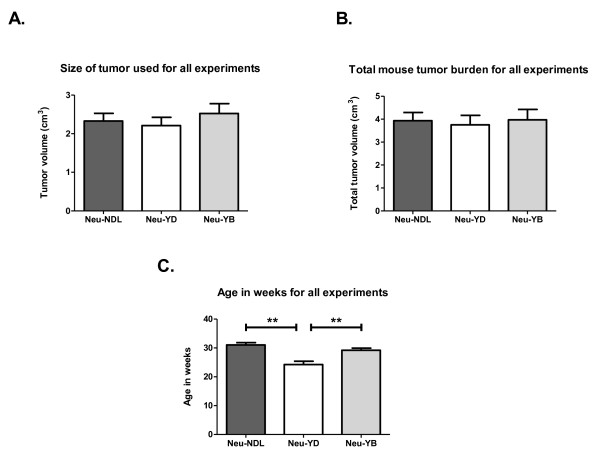
**Evaluation of tumor growth, burden and age**. **(A) **Size of largest tumor at time of analysis. There was no significant difference between the Neu deletion mutant (activated receptor) (Neu-NDL), Neu-YD and Neu-YB strains. **(B) **Total tumor volume at time of analysis. Transgenic mice developed multiple tumors. All tumors were measured, and the volumes were added. There was no significant difference in cumulative tumor volume, which averaged about 4 ml. **(C) **Average age in weeks at time of analysis. Data are means and SEM. **P < 0.005 by *t*-test, *n *= 18 to 22 mice per strain.

**Figure 2:**
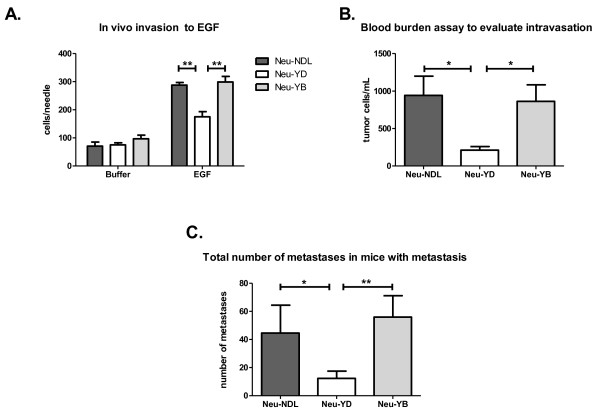
**Neu-YD strain exhibits decreased invasion, intravasation and metastasis compared to Neu-NDL and Neu-YB strains**. **(A) **The *in vivo *invasion assay was performed, allowing tumor cells to invade over 4 hours into microneedles containing 25 nM epidermal growth factor (EGF) or buffer. Neu-NDL = Neu deletion mutant (activated receptor). Data are means and SEM. **P < 0.005 by *t*-test, *n *= three to five mice per strain and five to seven needles per condition. **(B) **A blood burden assay was performed to evaluate intravasation. The number of single cells per milliliter of blood were counted. Data are means and SEM. *P < 0.05 by *t*-test, *n *= 8 to 11 mice per strain. **(C) **H & E-stained sections (5 μm each) spaced 250 μm apart were taken from the whole lung sample, and metastases were counted in every section. The efficiency of lung metastasis is represented as the total number of metastases in all lung sections for each animal that had metastases. Data are means and SEM. *P < 0.05 and ** p < 0.005 by Mann-Whitney *U *test; *n *= 16 Neu-NDL mice, 15 Neu-YD mice and 8 Neu-YB mice.

**Figure 3:**
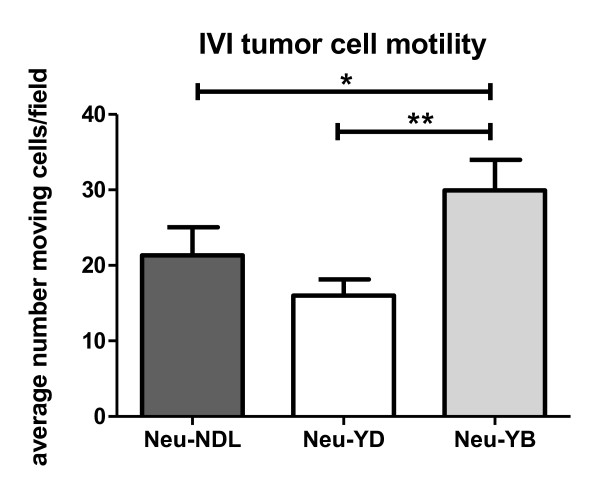
**Motility in the tumor microenvironment**. Intravital imaging (IVI) using multiphoton microscopy of primary mammary tumors from transgenic Neu deletion mutant (activated receptor) (Neu-NDL), Neu-YD and Neu-YB cyan fluorescent protein mice. Thirty-minute time-lapse Z-series were collected, and the average total cell motility was quantified per 50-μm Z-stack (five sections imaged at 10-μm intervals). Data are means and SEM of 27 to 32 separate Z-stacks. *P < 0.05, **P < 0.005 by Mann-Whitney *U *test; *n *= 13 Neu-NDL mice, *n *= 8 Neu-YD mice and *n *= 12 Neu-YB mice.

Metastatic properties were compared between animals with similar tumor volumes. At the time of analysis, the total tumor volume averaged 3 to 3.5 ml and the largest tumor averaged 2 to 2.5 ml (Figures 1A and 1B). Interestingly, the Neu-YD strain grew tumors the fastest, taking an average of 24 weeks compared to about 30 weeks for the Neu-NDL and Neu-YB strains (Figure 1C). Despite growing tumors faster (*P *< 0.005), the Neu-YD strain had decreased invasion, intravasation and metastasis (Figure 2). We evaluated invasion using the *in vivo *invasion assay previously described [34] by inserting microneedles containing EGF and Matrigel™ into the tumor to collect the invasive cell population. The Neu-YD tumor cells were significantly less invasive to EGF compared to those of the Neu-YB and the Neu-NDL strains (Figure 2A). To determine whether the decreased invasion of the Neu-YD strain correlated with a decreased number of tumor cells in the blood, we evaluated intravasation. The number of intravasated cells for the Neu-YD strain was approximately 200 cells/ml of blood. This number was significantly decreased compared to the Neu-YB strain, which averaged 850 cells/ml of blood, and the Neu-NDL strain, which averaged 940 cells/ml of blood (Figure 2B). The number of spontaneous metastases were counted in H & E sections throughout all lobes of the lungs in mice containing metastases. The Neu-YD strain had significantly fewer metastases than the Neu-YB or Neu-NDL strains (Figure 2C). This indicates the YB site, but not YD, is sufficient for lung metastasis. These results suggest that the defect in Neu-YD metastasis is due to defects in invasion and intravasation. To determine whether defects in invasion and intravasation were correlated with motility in the tumor microenvironment, we used intravital multiphoton microscopy to evaluate tumor cell motility *in vivo *(Figure 3 and Additional files 1, 2 and 3). We found that the Neu-YD tumors had slightly fewer motile cells than Neu-NDL. Neu-YB tumors had significantly more motile cells than either the Neu-NDL or Neu-YD tumors.

### Neu-YB strain has more macrophages than Neu-YD and Neu-NDL strains in tumor parenchyma

**Figure 4:**
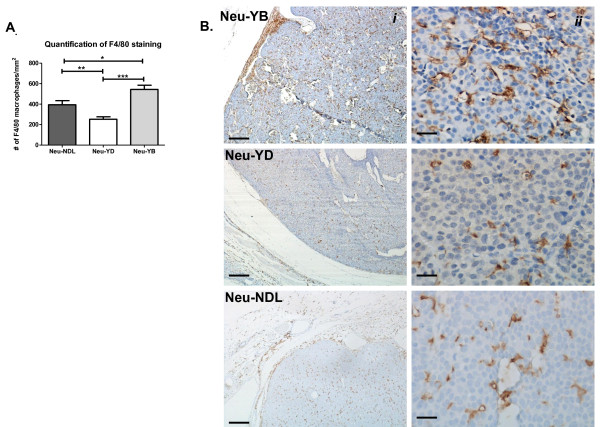
**F4/80 staining indicates the Neu-YB strain recruits more macrophages into the tumor parenchyma**. **(A) **F4/80 staining was quantified by using a 40× lens objective and counting the number of F4/80-stained macrophages per field (*n *= 3 tumors per strain and 10 random fields per tumor). Data shown are means and SEM. *P < 0.05, **P < 0.005, ***P < 0.0005 by *t*-test. **(B) **Representative fields of F4/80 staining taken at low magnification (scale bar = 200 μm) (i) and high magnification (scale bar = 25 μm) (ii). Neu-NDL = Neu deletion mutant (activated receptor).

In addition to our findings that the Neu-YB strain had increased invasion, intravasation, metastasis and motility compared to the Neu-YD strain, it has also been shown to be unique in its tumor morphology and expression profile compared to the other Neu lines [14,39]. Our past studies have been focused on the role of macrophages in a tumor cell and macrophage paracrine loop driving *in vivo *invasion [40,41]. We were interested to see whether differences in the number of macrophages in the tumor correlated with the differences we found in Neu-YB and Neu-YD *in vivo *invasion and tumor cell motility. We found that the Neu-YB tumors had, on average, twice the number of F4/80-positive macrophages per square millimeter compared to the Neu-YD tumors (*P *< 0.0005) and also had significantly more than the Neu-NDL control (*P *< 0.05) (Figures 4A and 4B). The Neu-YD strain also had fewer macrophages than the Neu-NDL (*P *< 0.005). In addition to macrophage recruitment, we looked at the distribution of vasculature and lymphatics in the various strains. We observed no difference in lymphatics (Additional file 4), but saw significantly more vasculature staining in the Neu-YB strain compared to the Neu-NDL strain (*P *< 0.05) and the Neu-YD strain (*P *< 0.0005) (Additional file 5). The Neu-YD strain also had significantly fewer vessels than the Neu-NDL strain (*P *< 0.005). The increase in vasculature seen in the Neu-YB strain is consistent with studies in which CXCL12 has been correlated with increased angiogenesis and hematopoiesis [23].

### EGF-induced *in vivo *invasion of Neu-YB tumor cells is dependent on CXCR4-CXC12 signaling in the tumor microenvironment

**Figure 5:**
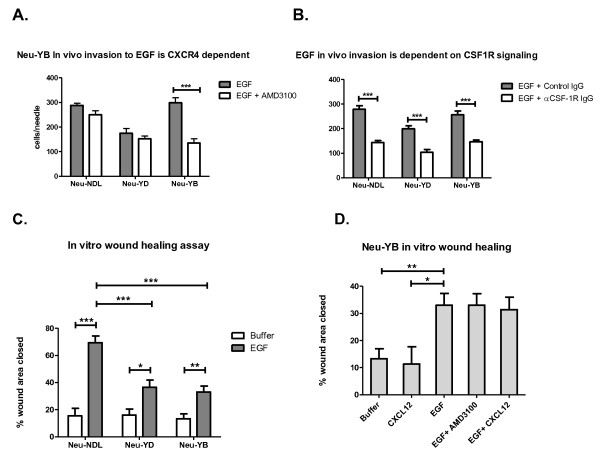
**Epidermal growth factor-induced *in vivo *invasion is dependent on CXCR4/CXCL12 paracrine signaling in the tumor microenvironment**. **(A) ***In vivo *invasion assay performed by infusing 25 nM epidermal growth factor (EGF) and EGF + 0.5 μM CXCR4 inhibitor AMD3100 through microneedles inserted into the primary tumor to collect the invasive cell population over 4 hours. Neu-NDL = Neu deletion mutant (activated receptor). Data are means and SEM. ***P < 0.0005 by *t*-test, *n *= three to five mice per strain and five to seven needles per condition. **(B) ***In vivo *invasion assay performed with 25 nM EGF + control rat immunoglobulin G (IgG) antibody or EGF + colony-stimulating factor 1 receptor (CSF-1R) blocking antibody (αCSF-1R IgG) in the needles. Data are means and SEM. ***P < 0.0005 by *t*-test, *n *= three to five mice per strain and five to seven needles per condition. **(C) ***In vitro *wound healing assays in the absence (Buffer) or presence of 5 nM EGF (EGF). Data are means and SEM. *P < 0.05, **P < 0.005 and ***P < 0.0005 by *t*-test, *n *= 3 tumors per strain and at least 10 fields per condition. **(D) ***In vitro *wound healing assays were performed with confluent primary tumor tissue in the presence of buffer (Buffer), 5 nM EGF alone (EGF), 5 nM EGF with 100 nM AMD3100 (EGF + AMD3100), 5 nM EGF with 1 nM CXCL12 (EGF + CXCL12) or 1 nM CXCL12 alone (CXCL12). Data are means and SEM. **P < 0.005 by *t*-test, *n *= 3 tumors and at least 10 fields per condition.

Because Neu-YB tumor cells have been shown to express more CXCL12 than the other mutants and Neu-NDL [14], we investigated whether CXCR4 signaling plays a role in EGF-induced *in vivo *invasion in that tumor. EGF-induced *in vivo *invasion of the Neu-YB strain was significantly inhibited by the CXCR4 inhibitor AMD3100 (*P *< 0.0005) (Figure 5A). The Neu-YD and Neu-NDL tumor cells were not significantly affected by AMD3100 inhibition of CXCR4. This suggests that the CXCL12 expressed by the Neu-YB tumor cells was necessary for EGF induced *in vivo *invasion. We have previously shown that EGF-induced *in vivo *invasion in the Neu-NDL model is dependent on a CSF-1-EGF paracrine loop [41]. To test this in the Neu-YB line, we used a CSF-1R-blocking antibody. The antibody inhibited EGF-induced *in vivo *invasion in the YB strain, as well as in the YD and Neu-NDL tumors, confirming that the paracrine loop was still operating (Figure 5B).

To evaluate *in vitro *migration, monolayers were grown from primary cells derived from the Neu-NDL, Neu-YD and Neu-YB primary tumors *in vitro*, and wound healing assays were carried out in the presence of EGF, EGF and AMD3100, and CXCL12. There was decreased EGF-induced *in vitro *migration in the Neu-YB and Neu-YD strains compared to the Neu-NDL in the wound healing assay (*P *< 0.0005) (Figure 5C). We evaluated CXCL12 secretion by ELISA in the primary cultures and found that the Neu-YB strain expressed more CXCL12 than the Neu-NDL and Neu-YD strains (Additional file 6, Supplemental Figure 3A). We confirmed that there were similar levels of CXCR4 receptor in all three tumor types (Additional file 6, Supplemental Figures 3B and 3C). However, there was no effect of CXCR4 inhibition or stimulation on *in vitro *wound healing in the Neu-YB strain (Figure 5D). The tumor cells did not respond to CXCL12 in a chemotaxis assay either (Additional file 6, Supplemental Figure 3D). Chemotaxis and wound healing assays with CXCL12 were also performed with MTLn3-CXCR4-overexpressing cells as positive controls (Additional file 6, Supplemental Figures 3D and 3E). The results of these studies indicate that the tumor cells are not activated through autocrine stimulation by CXCL12, but rather that a paracrine loop involving CXCR4-CXCL12 signaling in the tumor microenvironment is needed for EGF-induced *in vivo *invasion in Neu-YB tumors.

### Overexpression of CXCL12 in MTLn3 rat adenocarcinoma cells increases *in vivo *invasion and increases macrophage density in tumors

**Figure 6:**
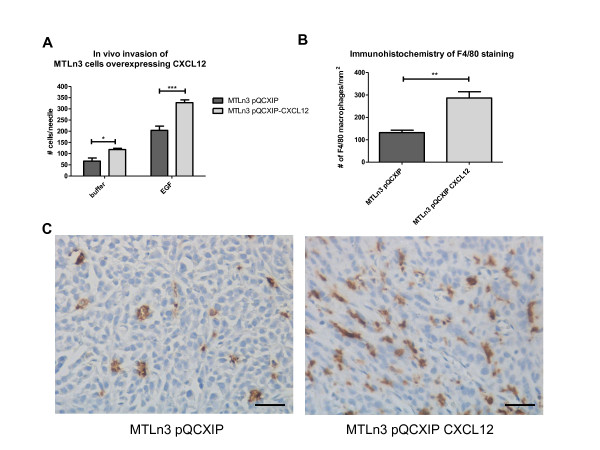
**Rat mammary adenocarcinoma CXCL12 cells show increased invasion to epidermal growth factor and recruitment of macrophages**. **(A) **The *in vivo *invasion assay was performed with MTLn3 empty vector and CXCL12-overexpressing cell line tumors, allowing tumor cells to invade over 4 hours into microneedles containing 25 nM EGF or buffer. Data shown are means and SEM. *P < 0.05 and ***P < 0.0005 by *t*-test. **(B) **F4/80 staining was quantified by using a 40× lens objective and counting the number of F4/80-stained macrophages per field (*n *= 5 tumors per cell line and 10 random fields per tumor). Data shown are means and SEM. **P < 0.005 by *t*-test. **(C) **Representative images of F4/80 staining for macrophages in MTLn3 pQCXIP and MTLn3 pQCXIP-CXCL12 tumors. Images were taken using a 40× lens objective. Scale bar = 25 μm.

To confirm that CXCL12 secretion by tumor cells can result in an increase in macrophage density and tumor cell *in vivo *invasion, we overexpressed CXCL12 in MTLn3 cells (Additional file 7). CXCL12 overexpression significantly increased *in vivo *invasion to EGF compared to the empty vector control (Figure 6A). Similarly to the Neu-YB tumors, CXCL12 overexpression in MTLn3 cells increased the number of macrophages present in the tumor parenchyma approximately twofold (*P *< 0.005) (Figures 6B and 6C). In addition, we evaluated CXCR4 expression levels on macrophages and tumor cells in the MTLn3 tumors and found that the macrophages in these tumors expressed more CXCR4 than the tumor cells did (Additional file 8, Supplemental Figure 5A). Of note, there was no significant difference in the amount of CXCR4 receptor on macrophages from the CXCL12-overexpressing tumors compared to the control (Additional file 8, Supplemental Figure 5A). Moreover, we found that CXCL12 induced the migration of CXCR4-positive macrophages (Additional file 8, Supplemental Figures 5B and 5C). CXCL12 overexpression had no effect on tumor size. However, we did observe a trend toward increased numbers of metastases with CXCL12 expression (Additional file 9, Supplemental Figures 6A and 6B). Consistent with our observation that the Neu-YB tumors, which express increased amounts of CXCL12, have a higher microvessel density, we confirmed that CXCL12 expression in MTLn3 cells also significantly increases the number of blood vessels in the primary tumors compared to control MTLn3 tumors (Additional file 9, Supplemental Figures 6C through 6E).

## Discussion

In this paper, we describe how the expression of CXCL12 by breast tumor cells can enhance *in vivo *invasion in the tumor microenvironment through recruitment of macrophages. We initially analyzed the Neu-transgenic tumors. The Neu-YB and Neu-YD strains had different metastatic capacities, which is consistent with previously published data [14]. We found that the Neu-YD tumor cells are defective in wound healing *in vitro *and that this correlates with decreased *in vivo *invasion in the primary tumor and decreased metastasis to the lungs. The Neu-YB tumor cells showed a reduction in wound healing *in vitro *similar to that of the Neu-YD cells. *In vivo*, however, they exhibited increased motility compared to both the Neu-NDL and Neu-YD strains and increased *in vivo *invasion, intravasation and metastasis compared to the Neu-YD strain. The increased *in vivo *invasion was dependent on CXCR4 function, which is consistent with the overexpression of CXCL12 by Neu-YB tumors. We then directly tested the role of CXCL12 expression in tumor cells by overexpressing this ligand in MTLn3 cells. Increased CXCL12 expression in MTLn3 cells resulted in increased *in vivo *invasion, macrophage density and microvessel numbers, which is consistent with our observations of Neu-YB tumors.

The CXCR4-CXCL12 signaling axis contributes to metastasis and clinical outcomes in breast cancer. CXCR4 expression in breast cancer cells has been shown to increase metastasis by homing of tumor cells to sites of increased CXCL12 expression, such as the lymph nodes [16]. Overexpression of CXCL12 increased *in vitro *invasion and migration of human breast cancer MDA-MB-231 cells [25], and overexpression of CXCL12 was seen in breast cancer patients with lymph node-positive metastatic disease with a poor prognosis [25]. We have directly evaluated the role of increased CXCL12 expression in *in vivo *tumor cell invasion, where the tumor microenvironment and paracrine loops with supporting stromal cells can influence tumor cell behavior. We found that inhibiting CXCR4 with AMD3100 reduced Neu-YB *in vivo *invasion to EGF to levels similar to those seen in Neu-YD. This indicates that invasion to EGF is dependent on CXCL12-CXCR4 signaling in the tumor microenvironment, specifically in the Neu-YB strain.

We also saw increased numbers of macrophages in the Neu-YB tumor parenchyma. Because there is an EGF-CSF-1R paracrine loop with macrophages inducing tumor cell invasion, we tested whether CXCL12 would act on the Neu-YB tumor cells to increase invasion in an autocrine manner or in a paracrine loop with macrophages. As we have shown previously for the EGF-CSF1 paracrine loop [40], a blocking antibody to the CSF-1R decreased *in vivo *invasion in the YB strain, indicating that *in vivo *invasion of the YB tumor cells was still dependent upon the EGFR-CSF-1R paracrine loop interaction with macrophages. We found that, *in vitro*, both Neu-YB and Neu-YD primary tumor cells had impaired wound healing to EGF compared to the Neu-NDL cells. Addition of CXCL12 did not increase *in vitro *wound healing in the Neu-YB cultures, and AMD3100 did not inhibit it, supporting the hypothesis that in this model CXCL12 contributes to a paracrine loop between tumor cells and macrophages within the tumor microenvironment. We hypothesize that the increased macrophage recruitment in the Neu-YB tumors results in enhanced paracrine loop invasion, which compensates for the reduced inherent invasiveness in the Neu-YB cells. Thus the inherent defect in Neu-YB tumor cell invasiveness is compensated by increased CXCL12 expression. To confirm that CXCL12 expression can produce such effects, we overexpressed CXCL12 in MTLn3 breast cancer cells and observed increased *in vivo *invasion as well as increased tumor-associated macrophage recruitment.

There are a number of possible mechanisms by which increased CXCL12 expression could enhance tumor invasiveness and malignancy. The results of the present study indicate that CXCL12 overexpression itself, in different tumor models, can recruit more macrophages. The increased recruitment of tumor-associated macrophages may induce increased tumor cell motility and invasion simply by increasing the number of macrophages that contribute to paracrine loop signaling [40,42-45]. In addition, secreted CXCL12 signaling to CXCR4 receptors on macrophages can increase EGFR ligand shedding [46], and this could also contribute to increased tumor cell motility and invasion. Alternatively, in tumors expressing CXCR4, autocrine signaling has been shown to increase migration and invasion in other models [25,47]. CXCL12 can transactivate the EGFR and Neu through Src activation [43,48], and EGF can potentiate CXCL12-driven chemotaxis [49]. Our *in vitro *studies showing that Neu-YB cells are similar to Neu-YD cells in the wound healing *in vitro *assay, with the addition of CXCL12 or inhibition of CXCR4 having no effect, argue against autocrine signaling's contributing to the invasion of Neu-YB tumor cells.

In this study, we also found increased microvessel density in tumors that overexpressed CXCL12. Because the tumor cells we studied did not show strong responsiveness to CXCL12, the increased microvessel density in tumors that overexpressed CXCL12 most likely reflects a stromal response to CXCL12. In particular, the increased macrophage density may also give rise to increased angiogenesis through secretion of angiogenic factors such as vascular endothelial growth factor [50-53]. Intriguingly, we did not find that increased microvessel density was correlated with enhanced tumor growth. However, the increased microvessel density could result in increased intravasation through the presence of a higher density of entrance sites into the blood, with a corresponding increase in the formation of metastases.

## Conclusion

In summary, we have found that secretion of CXCL12 by breast cancer cells can enhance invasion *in vivo *and recruitment of macrophages to the primary tumor. This enhancement of invasion depends upon CXCR4 signaling and most likely occurs through activation of CXCR4 on macrophages, resulting in increased paracrine interactions with tumor cells in the tumor microenvironment. Increased CXCL12 secretion also gives rise to increased microvessel density, which may also be mediated by tumor-associated macrophages, and contributes to altered tumor architecture. These results demonstrate how tumor cell regulation of the local cellular microenvironment can increase invasion and motility and thus contribute to enhanced tumor malignancy.

## Abbreviations

BMM: bone marrow-derived macrophage; BSA: bovine serum albumin; CFP: cyan fluorescent protein; CSF-1R: colony-stimulating factor 1 receptor; DMEM: Dulbecco's modified Eagle's medium; EGF: epidermal growth factor; ELISA: enzyme-linked immunosorbent assay; FBS: fetal bovine serum; GFP: green fluorescent protein; H & E: hematoxylin and eosin; LYVE-1: lymphatic vessel endothelial hyaluronan receptor; mAb: monoclonal antibody; MMTV: mouse mammary tumor virus; MTLn3: rat mammary adenocarcinoma cell line; Neu-NDL: Neu deletion mutant (activated receptor); pAb: polyclonal antibody; PBS: phosphate-buffered saline; PLPG: periodate-lysine-2% paraformaldehyde-0.05% glutaraldehyde; RT-PCR: reverse transcriptase polymerase chain reaction.

## Competing interests

The authors declare that they have no competing interests.

## Authors' contributions

PB and TS contributed equally to carrying out *in vivo *and *in vitro *experiments, analyzing data, performing multiphoton imaging and analysis, and preparing the manuscript. PB bred the transgenic mice and performed *in vivo *studies in severe combined immunodeficient mice. ZZ carried out additional immunohistochemistry studies for tumor vasculature, as well as additional *in vitro *wound healing assays and MTLn3 wound healing and chemotaxis assays. JW helped to carry out multiphoton imaging and contributed intellectually to the development of this study. JC contributed intellectually to the development of this study and kindly provided reagents. HP and DC carried out *in vitro *BMM chemotaxis studies and BMM immunofluorescence. SC performed and analyzed quantitative RT-PCR. JWP provided assistance in breeding transgenic mice and fluorescence-activated cell sorting (FACS) analysis. WJM provided the Neu-NDL, Neu-YB and Neu-YD mice. BZQ performed FACS analysis during the development of the study and contributed to its intellectual content. ERS provided the PLPG fixation protocol and reagents. JES contributed intellectually to the development and initiation of the study and assisted in preparing the manuscript. All authors read and approved the final manuscript for publication.

## Supplementary Material

Additional file 1 **Supplemental movies 1, 2 and 3**. The Neu-YB (Additional file 3) mouse strain tumors had more motility than the Neu deletion mutant (activated receptor) (Neu-NDL) (Additional file 1) or Neu-YD (Additional file 2) mouse strains in the tumor microenvironment. Tumors were exposed by performing skin flap surgery, and the murine tumor tissue was imaged with an Olympus FluoView FV1000-MPE multiphoton laser scanning microscope. Cyan fluorescent protein-expressing tumors were excited at a wavelength of 880 nm using a 25× 1.05-numerical aperture water immersion lens objective with a 2.3× zoom. Time-lapse imaging was done over 30 minutes, collecting a 3D image stack through 100 μm every 2 minutes in 5 μm steps in the z axis. Arrows indicate areas of cell motility. Scale bar = 25 μm. Click here for file

Additional file 2 **Supplemental movies 1, 2 and 3**. The Neu-YB (Additional file 3) mouse strain tumors had more motility than the Neu deletion mutant (activated receptor) (Neu-NDL) (Additional file 1) or Neu-YD (Additional file 2) mouse strains in the tumor microenvironment. Tumors were exposed by performing skin flap surgery, and the murine tumor tissue was imaged with an Olympus FluoView FV1000-MPE multiphoton laser scanning microscope. Cyan fluorescent protein-expressing tumors were excited at a wavelength of 880 nm using a 25× 1.05-numerical aperture water immersion lens objective with a 2.3× zoom. Time-lapse imaging was done over 30 minutes, collecting a 3D image stack through 100 μm every 2 minutes in 5 μm steps in the z axis. Arrows indicate areas of cell motility. Scale bar = 25 μm. Click here for file

Additional files 3 **Supplemental movies 1, 2 and 3**. The Neu-YB (Additional file 3) mouse strain tumors had more motility than the Neu deletion mutant (activated receptor) (Neu-NDL) (Additional file 1) or Neu-YD (Additional file 2) mouse strains in the tumor microenvironment. Tumors were exposed by performing skin flap surgery, and the murine tumor tissue was imaged with an Olympus FluoView FV1000-MPE multiphoton laser scanning microscope. Cyan fluorescent protein-expressing tumors were excited at a wavelength of 880 nm using a 25 × 1.05-numerical aperture water immersion lens objective with a 2.3× zoom. Time-lapse imaging was done over 30 minutes, collecting a 3D image stack through 100 μm every 2 minutes in 5 μm steps in the z axis. Arrows indicate areas of cell motility. Scale bar = 25 μm. Click here for file

Additional file 4 **Supplemental Figure 1 Immunohistochemistry indicates no difference in density of lymphatic vessels in the Neu primary tumors**. Tumors from the Neu deletion mutant (activated receptor) (Neu-NDL), Neu-YD and Neu-YB mice were fixed in 10% buffered formalin, then sectioned and stained using the lymphatic vessel endothelial hyaluronan receptor (LYVE-1) antibody against mouse LYVE-1 to stain lymphatic endothelial cells. Representative images of each stain are shown. Scale bar = 100 μm. Click here for file

Additional file 5 **Supplemental Figure 2 Neu-YB tumors show increased vasculature**. Tumors from the Neu deletion mutant (activated receptor) (Neu-NDL), Neu-YD and Neu-YB strains were fixed in 10% buffered formalin, then sectioned and stained using an endomucin antibody to detect vasculature. **(A) **Vessels were quantified using a 20× lens objective. Ten random fields per tumor were counted (*n *= three tumors per strain). Data are means and SEM. *P < 0.05, **P < 0.005 and ***P < 0.0005. **(B) **Representative images of each strain are shown. Scale bar = 100 μm. Click here for file

Additional file 6 **Supplemental Figure 3 Further characterization of CXCL12 and CXCR4 in the Neu tumors**. **(A) **Primary culture cell lines were counted and seeded in duplicate and supernatants were collected for CXCL12 quantification 16 hours after cultures were confluent. ELISA was done in triplicate for each sample using the CXCL12 mouse ELISA from R&D Systems. Data are means and SEM. **(B) **Tumors from the Neu deletion mutant (activated receptor) (Neu-NDL), Neu-YD and Neu-YB strains were fixed in 10% buffered formalin, then sectioned and stained using anti-CXCR4 antibody. Representative images for each strain are shown. Scale bar = 50 μm. **(C) **mRNA was extracted from the Neu primary tumor cells in culture. Levels of CXCR4 are represented as average change in threshold cycle values normalized to Neu-NDL (*n *= three samples per strain). Data are means and SEM. **(D) **CXCL12-induced chemotaxis of primary tumor cells (top) or mammary adenocarcinoma (MTLn3) CXCR4-overexpressing cells (bottom) was determined using a microchemotaxis Boyden chamber assay. Data are means and SEM. **(E) ***In vitro *wound healing assay with MTLn3 CXCR4 cells in the absence (Buffer) or presence of 1 nM CXCL12. Data are means and SEM, *N *= 3, 10 fields per condition. *P < 0.05. Click here for file

Additional file 7 **Supplemental Figure 4 Mammary adenocarcinoma GFP CXCL12 cell line overexpressed CXCL12 compared to empty vector control cells**. Mammary adenocarcinoma (MTLn3) GFP CXCL12 and MTLn3 GFP pQCXIP control cell lines were plated in triplicate, and supernatants were collected from confluent cultures after 16 hours. Cells were counted to normalize for cell number, and ELISA was performed in triplicate for each sample using the CXCL12 mouse ELISA from R&D Systems. Data are means and SEM. ***P < 0.0005. Click here for file

Additional file 8 **Supplemental Figure 5 Macrophage CXCR4 expression and responses to CXCL12**. **(A) **Expression of CXCR4 on tumor cells and macrophages *in vivo*. Mammary adenocarcinoma tumors formed from MTLn3 GFP cells stably transduced with pQCXIP-CXCL12 or pQCXIP empty vector control were dissociated and labeled with CD45-AF700, F4/80-PerCP and CXCR4-PE antibodies, respectively. Cells were washed and stained with 4',6-diamidino-2-phenylindole as a viability marker. Samples were analyzed using flow cytometry, and data were processed using FlowJo software. Tumor cells were CD45-, F4/80- and GFP+. Macrophages were CD45+ and F4/80+. The mean fluorescence of CXCR4-phycoerythrin is shown in representative images (*n *= three tumors for each cell line). **(B) **Expression of CXCR4 on macrophages *in vitro*. Representative images of live murine bone marrow-derived macrophages (BMMs) stained for control or CXCR4 (right panels). Matched phase-contrast images are shown (left panels). Scale bar = 10 μm. **(C) **Chemotaxis of macrophages *in vitro*. CXCL12-induced chemotaxis of BMMs with 50 ng/ml CXCL12 was determined using a transwell assay and is expressed as cells per field (*n *= 3). Data are means and SEM. ***P < 0.0003. Click here for file

Additional file 9 **Supplemental Figure 6 Tumor volume, lung metastasis and vasculature of mammary adenocarcinoma GFP CXCL12 tumors**. **(A) **Tumor volume was calculated by measuring the length and width of each tumor. There were no significant differences in tumor volume between the CXCL12 overexpressors and empty vector controls. **(B) **Lung metastasis was quantified as the total number of micrometastases in all lobes per section stained by H & E. The mammary adenocarcinoma (MTLn3) CXCL12 tumors displayed a trend toward increased metastases. Error bars = SEM (*n *= ten mice). **(C) **MTLn3 empty vector control and MTLn3 CXCL12 tumors were fixed in 10% buffered formalin, then sectioned and stained using endomucin antibody against the mouse endothelial cells to stain vasculature. Vessels were quantified using a 20× lens objective. Ten random fields per tumor were counted (*n *= three tumors per strain). Data are means and SEM. ***P < 0.0002. **(D) **and **(E) **Representative images of the control tumors (D) and CXCL12 tumors (E) are shown. Scale bar = 100 μm. Click here for file
