# Supplementary figures and images for: Contribution of CXCL12 secretion to invasion of breast cancer cells
Source: Breast Cancer Res. 2012 Feb 7;14(1):R23. doi: 10.1186/bcr3108 (PMC3496141; doi:10.1186/bcr3108)

## Slide 1
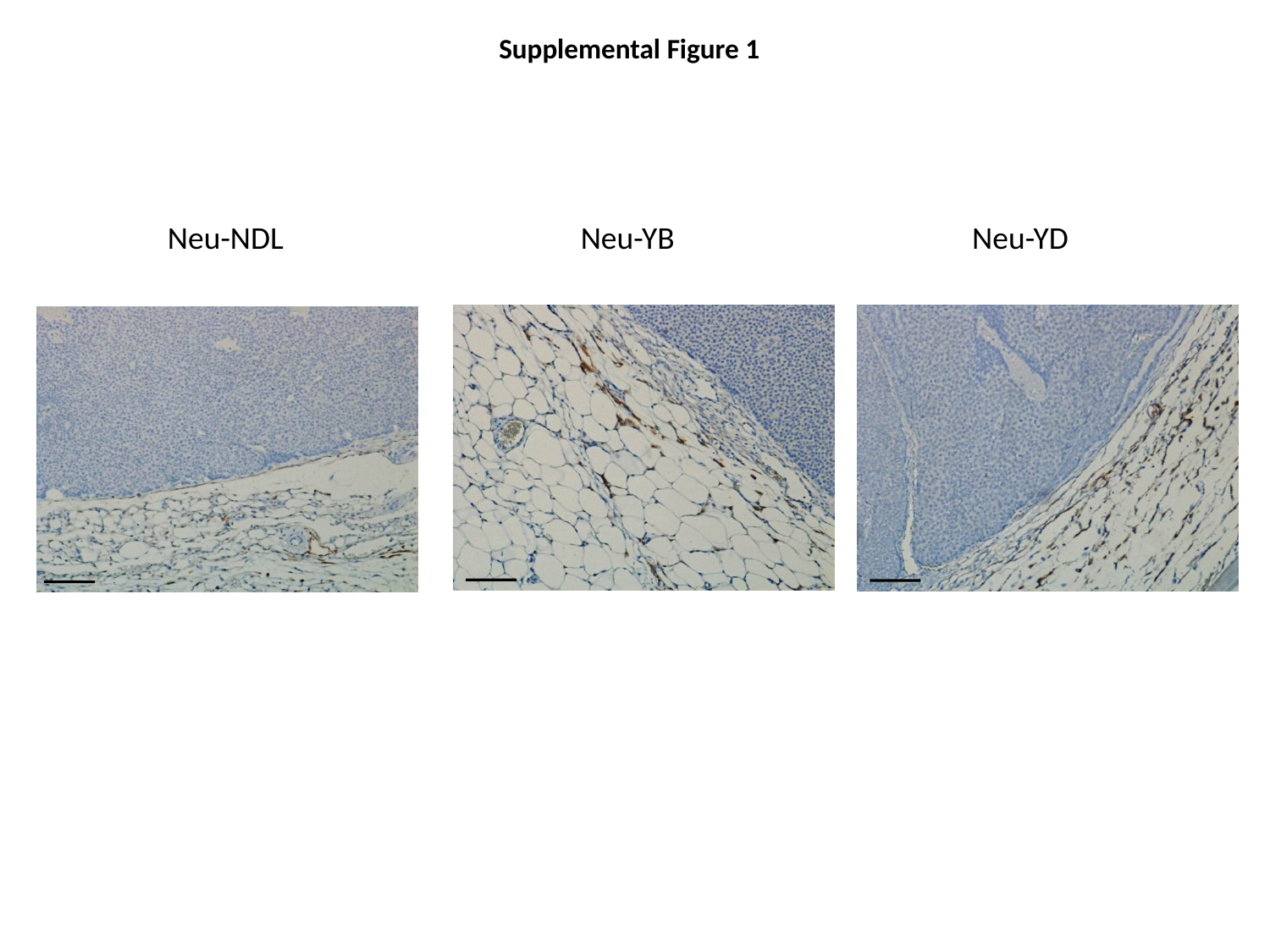

Supplemental Figure 1
Neu-NDL
Neu-YB
Neu-YD

Supplement: Additional file 4 — Supplemental Figure 1 Immunohistochemistry indicates no difference in density of lymphatic vessels in the Neu primary tumors. Tumors from the Neu deletion mutant (activated receptor) (Neu-NDL), Neu-YD and Neu-YB mice were fixed in 10% buffered formalin, then sectioned and stained using the lymphatic vessel endothelial hyaluronan receptor (LYVE-1) antibody against mouse LYVE-1 to stain lymphatic endothelial cells. Representative images of each stain are shown. Scale bar = 100 μm. [file bcr3108-S4.PPT]

## Slide 1
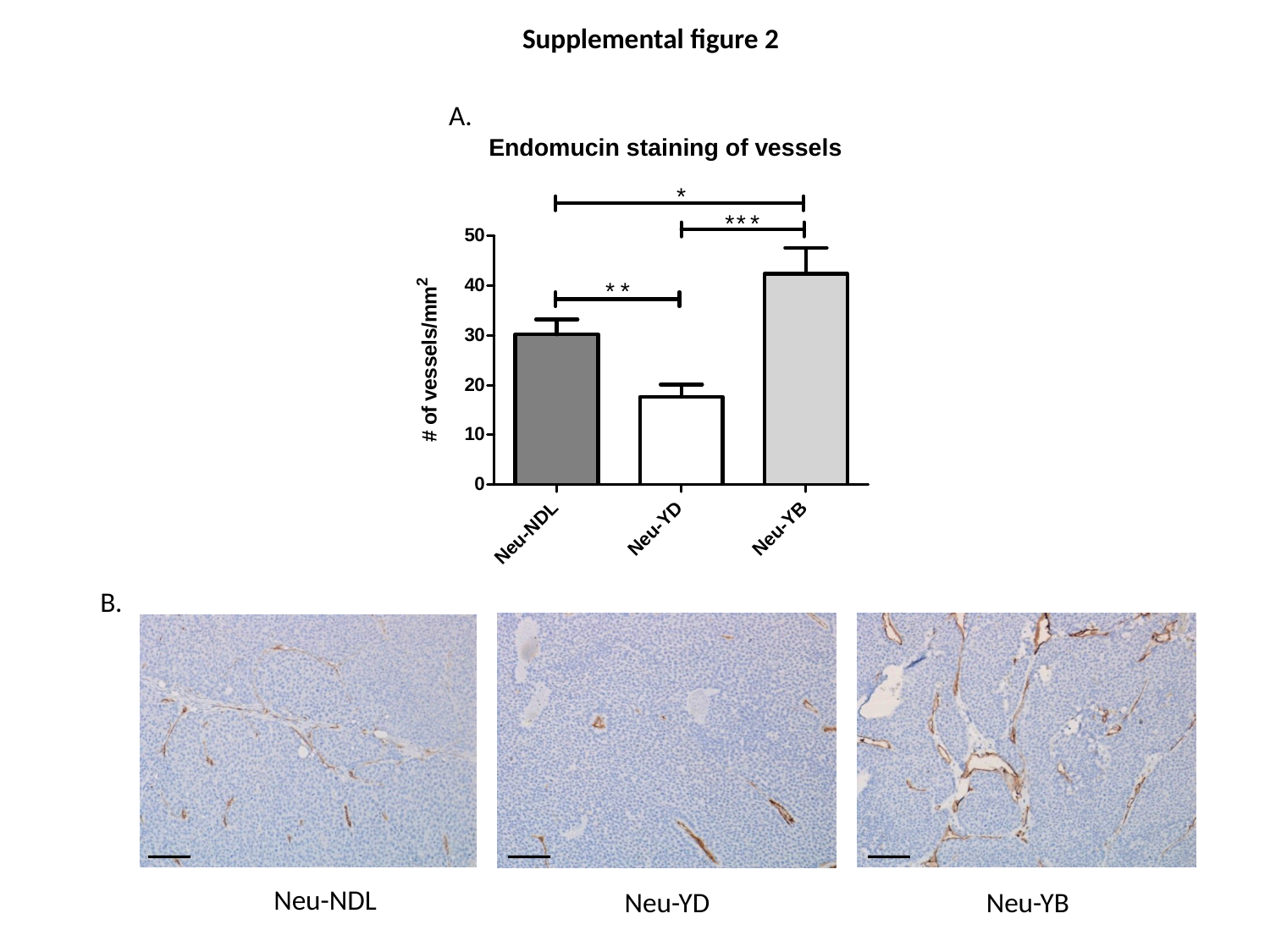

# Supplemental figure 2
A.
B.
Neu-NDL
Neu-YD
Neu-YB

Supplement: Additional file 5 — Supplemental Figure 2 Neu-YB tumors show increased vasculature. Tumors from the Neu deletion mutant (activated receptor) (Neu-NDL), Neu-YD and Neu-YB strains were fixed in 10% buffered formalin, then sectioned and stained using an endomucin antibody to detect vasculature. (A) Vessels were quantified using a 20× lens objective. Ten random fields per tumor were counted (n = three tumors per strain). Data are means and SEM. *P < 0.05, **P < 0.005 and ***P < 0.0005. (B) Representative images of each strain are shown. Scale bar = 100 μm. [file bcr3108-S5.PPT]

## Slide 1
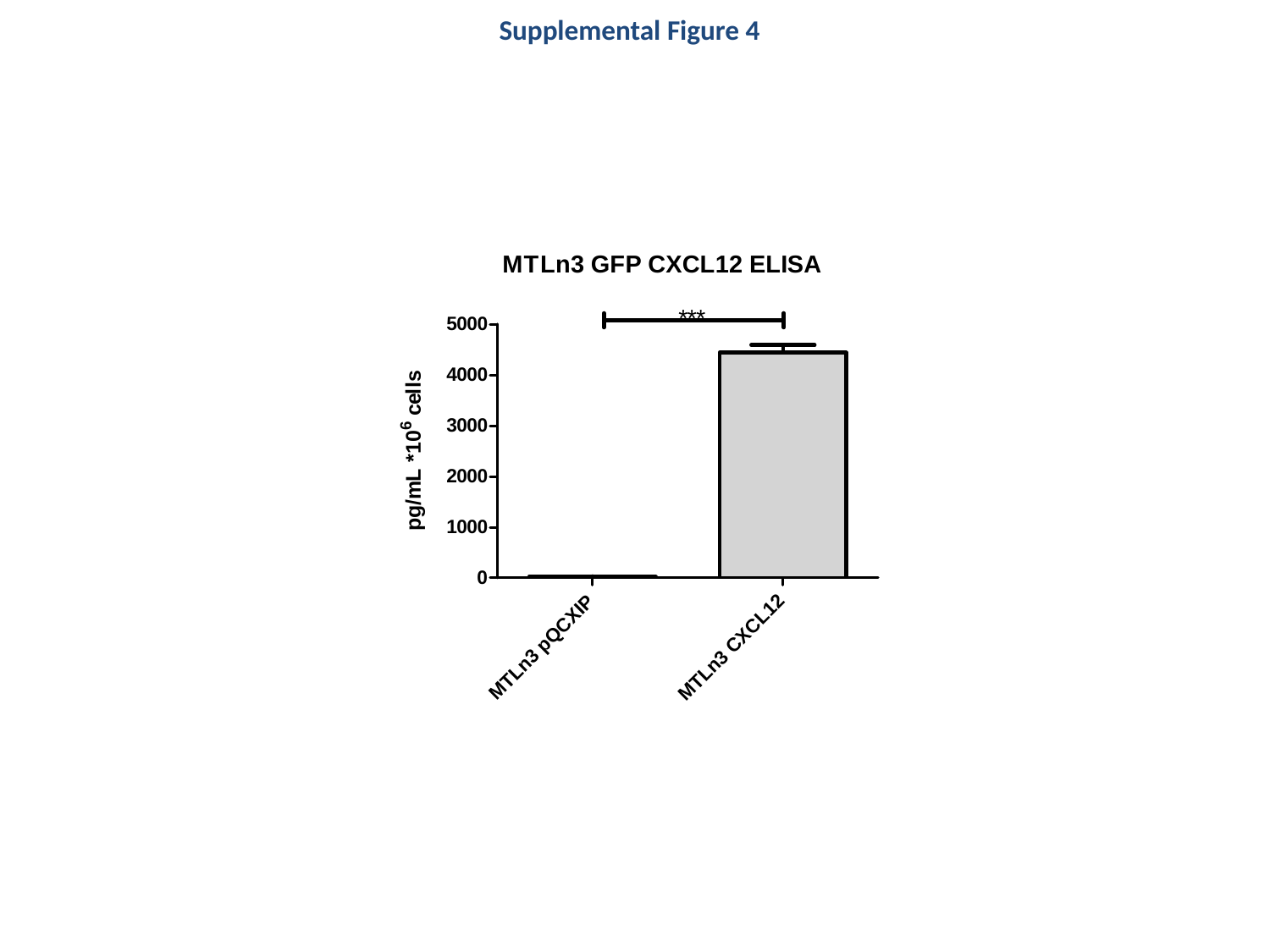

Supplemental Figure 4

Supplement: Additional file 7 — Supplemental Figure 4 Mammary adenocarcinoma GFP CXCL12 cell line overexpressed CXCL12 compared to empty vector control cells. Mammary adenocarcinoma (MTLn3) GFP CXCL12 and MTLn3 GFP pQCXIP control cell lines were plated in triplicate, and supernatants were collected from confluent cultures after 16 hours. Cells were counted to normalize for cell number, and ELISA was performed in triplicate for each sample using the CXCL12 mouse ELISA from R&D Systems. Data are means and SEM. ***P < 0.0005. [file bcr3108-S7.PPT]

## Slide 1
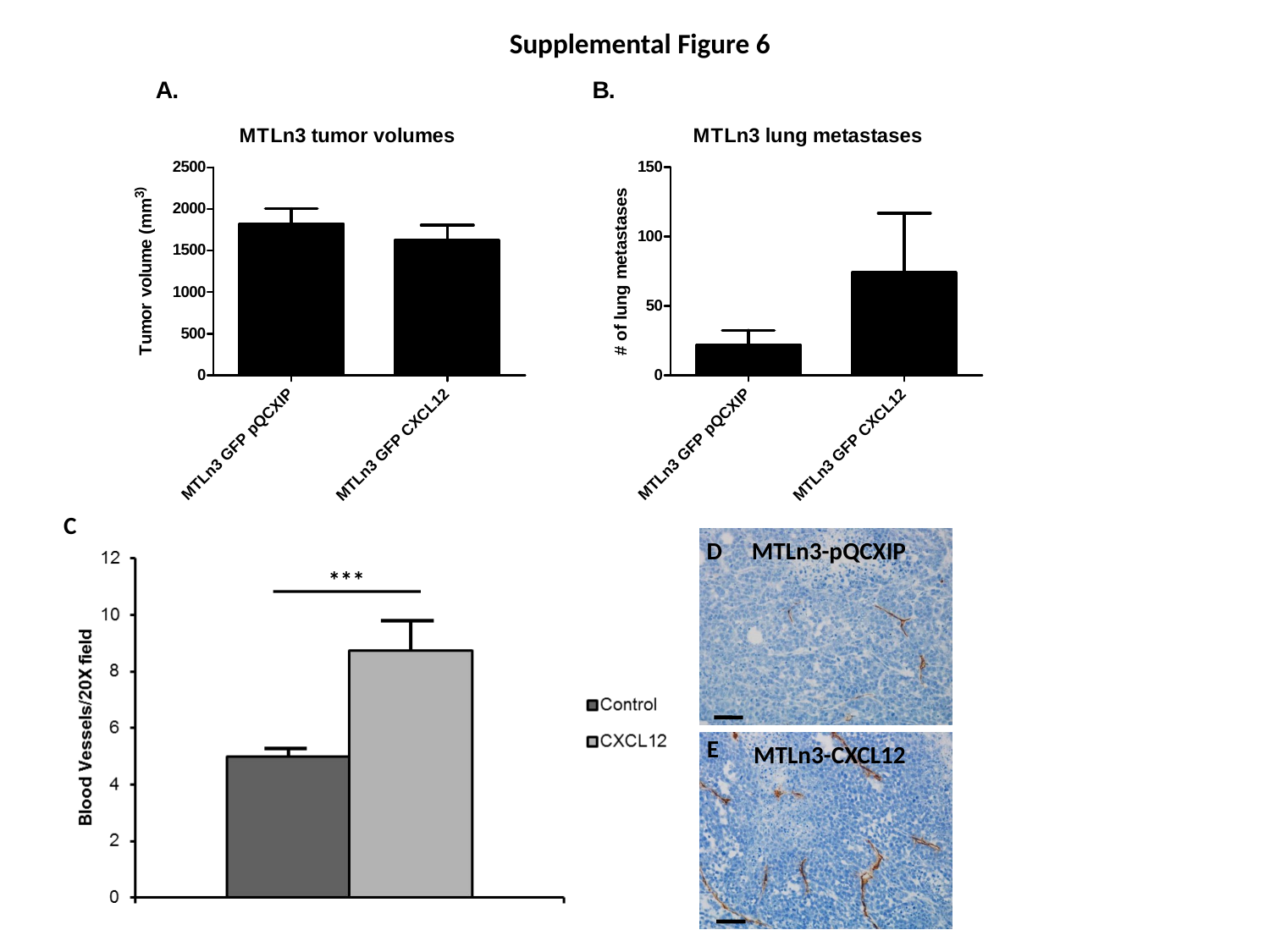

Supplemental Figure 6
C
***
D
MTLn3-pQCXIP
E
MTLn3-CXCL12

Supplement: Additional file 9 — Supplemental Figure 6 Tumor volume, lung metastasis and vasculature of mammary adenocarcinoma GFP CXCL12 tumors. (A) Tumor volume was calculated by measuring the length and width of each tumor. There were no significant differences in tumor volume between the CXCL12 overexpressors and empty vector controls. (B) Lung metastasis was quantified as the total number of micrometastases in all lobes per section stained by H & E. The mammary adenocarcinoma (MTLn3) CXCL12 tumors displayed a trend toward increased metastases. Error bars = SEM (n = ten mice). (C) MTLn3 empty vector control and MTLn3 CXCL12 tumors were fixed in 10% buffered formalin, then sectioned and stained using endomucin antibody against the mouse endothelial cells to stain vasculature. Vessels were quantified using a 20× lens objective. Ten random fields per tumor were counted (n = three tumors per strain). Data are means and SEM. ***P < 0.0002. (D) and (E) Representative images of the control tumors (D) and CXCL12 tumors (E) are shown. Scale bar = 100 μm. [file bcr3108-S9.PPT]
