# Supplementary material for: Contribution of CXCL12 secretion to invasion of breast cancer cells
Source: Breast Cancer Res. 2012 Feb 7;14(1):R23. doi: 10.1186/bcr3108 (PMC3496141; doi:10.1186/bcr3108)
Supplement: Additional file 6 — Supplemental Figure 3 Further characterization of CXCL12 and CXCR4 in the Neu tumors. (A) Primary culture cell lines were counted and seeded in duplicate and supernatants were collected for CXCL12 quantification 16 hours after cultures were confluent. ELISA was done in triplicate for each sample using the CXCL12 mouse ELISA from R&D Systems. Data are means and SEM. (B) Tumors from the Neu deletion mutant (activated receptor) (Neu-NDL), Neu-YD and Neu-YB strains were fixed in 10% buffered formalin, then sectioned and stained using anti-CXCR4 antibody. Representative images for each strain are shown. Scale bar = 50 μm. (C) mRNA was extracted from the Neu primary tumor cells in culture. Levels of CXCR4 are represented as average change in threshold cycle values normalized to Neu-NDL (n = three samples per strain). Data are means and SEM. (D) CXCL12-induced chemotaxis of primary tumor cells (top) or mammary adenocarcinoma (MTLn3) CXCR4-overexpressing cells (bottom) was determined using a microchemotaxis Boyden chamber assay. Data are means and SEM. (E) In vitro wound healing assay with MTLn3 CXCR4 cells in the absence (Buffer) or presence of 1 nM CXCL12. Data are means and SEM, N = 3, 10 fields per condition. *P < 0.05. [file bcr3108-S6.PPT]

## Slide 1
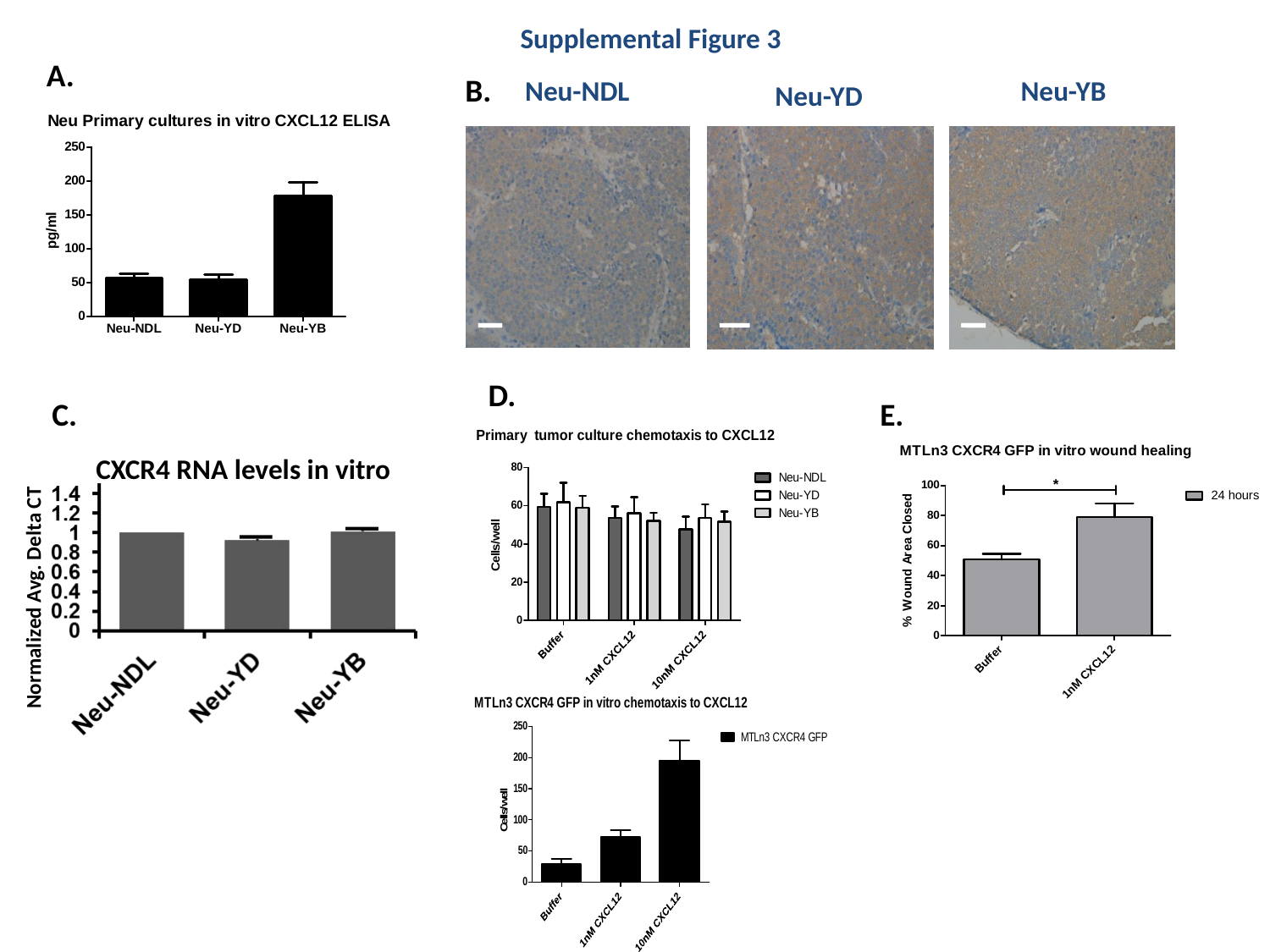

Supplemental Figure 3
A.
Neu-NDL
Neu-YB
Neu-YD
B.
D.
C.
E.
CXCR4 RNA levels in vitro
Normalized Avg. Delta CT
