# Supplementary material for: Contribution of CXCL12 secretion to invasion of breast cancer cells
Source: Breast Cancer Res. 2012 Feb 7;14(1):R23. doi: 10.1186/bcr3108 (PMC3496141; doi:10.1186/bcr3108)
Supplement: Additional file 8 — Supplemental Figure 5 Macrophage CXCR4 expression and responses to CXCL12. (A) Expression of CXCR4 on tumor cells and macrophages in vivo. Mammary adenocarcinoma tumors formed from MTLn3 GFP cells stably transduced with pQCXIP-CXCL12 or pQCXIP empty vector control were dissociated and labeled with CD45-AF700, F4/80-PerCP and CXCR4-PE antibodies, respectively. Cells were washed and stained with 4',6-diamidino-2-phenylindole as a viability marker. Samples were analyzed using flow cytometry, and data were processed using FlowJo software. Tumor cells were CD45-, F4/80- and GFP+. Macrophages were CD45+ and F4/80+. The mean fluorescence of CXCR4-phycoerythrin is shown in representative images (n = three tumors for each cell line). (B) Expression of CXCR4 on macrophages in vitro. Representative images of live murine bone marrow-derived macrophages (BMMs) stained for control or CXCR4 (right panels). Matched phase-contrast images are shown (left panels). Scale bar = 10 μm. (C) Chemotaxis of macrophages in vitro. CXCL12-induced chemotaxis of BMMs with 50 ng/ml CXCL12 was determined using a transwell assay and is expressed as cells per field (n = 3). Data are means and SEM. ***P < 0.0003. [file bcr3108-S8.PPT]

## Slide 1
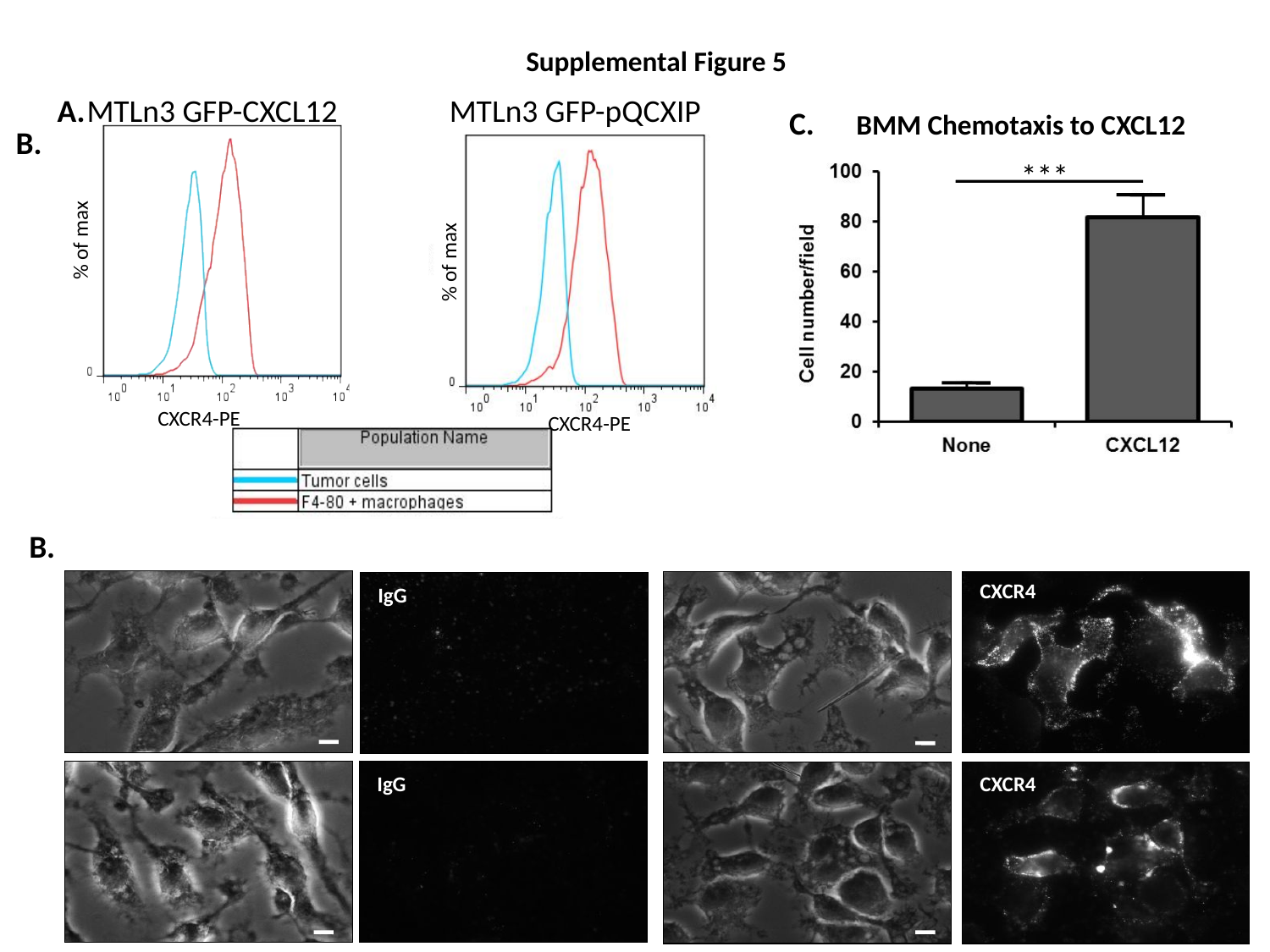

Supplemental Figure 5
A.
MTLn3 GFP-CXCL12
MTLn3 GFP-pQCXIP
C.
BMM Chemotaxis to CXCL12
B.
***
% of max
% of max
CXCR4-PE
CXCR4-PE
B.
CXCR4
IgG
IgG
CXCR4
